# Hypothalamic Transcriptome Analysis Reveals the Crucial MicroRNAs and mRNAs Affecting Litter Size in Goats

**DOI:** 10.3389/fvets.2021.747100

**Published:** 2021-11-01

**Authors:** Chen Liang, Miaoceng Han, Zuyang Zhou, Yufang Liu, Xiaoyun He, Yanting Jiang, Yina Ouyang, Qionghua Hong, Mingxing Chu

**Affiliations:** ^1^College of Animal Science, Shanxi Agricultural University, Taigu, China; ^2^Key Laboratory of Animal Genetics, Breeding and Reproduction of Ministry of Agriculture and Rural Affairs, Institute of Animal Science, Chinese Academy of Agricultural Sciences (CAAS), Beijing, China; ^3^Yunnan Animal Science and Veterinary Institute, Kunming, China

**Keywords:** goats, reproduction, hypothalamus, mRNAs, miRNAs

## Abstract

The hypothalamus was the coordination center of the endocrine system, which played an important role in goat reproduction. However, the molecular mechanism of hypothalamus regulating litter size in goats was still poorly understood. This study aims to investigate the key functional genes associated with prolificacy by hypothalamus transcriptome analysis of goats. In this research, an integrated analysis of microRNAs (miRNAs)-mRNA was conducted using the hypothalamic tissue of Yunshang black goats in the follicular stage. A total of 72,220 transcripts were detected in RNA-seq. Besides, 1,836 differentially expressed genes (DEGs) were identified between high fecundity goats at the follicular phase (FP-HY) and low fecundity goats at the follicular phase (FP-LY). DEGs were significantly enriched in 71 Gene Ontology (GO) terms and 8 Kyoto Encyclopedia of Genes and Genomes (KEGG) pathways. The transcriptome data suggested that DEGs such as *BMPR1B, FGFR1, IGF1* and *CREB1* are directly or indirectly involved in many processes like hypothalamic gonadal hormone secretion. The miRNA-seq identified 1,837 miRNAs, of which 28 differentially expressed miRNAs (DEMs). These DEMs may affect the nerve cells survival of goat hypothalamic regulating the function of target genes and further affect the hormone secretion activities related to reproduction. They were enriched in prolactin signaling pathway, Jak-STAT signaling pathway and GnRH signaling pathway, as well as various metabolic pathways. Integrated analysis of DEMs and DEGs showed that 87 DEGs were potential target genes of 28 DEMs. After constructing a miRNA-mRNA pathway network, we identified several mRNA-miRNAs pairs by functional enrichment analysis, which was involved in hypothalamic nerve apoptosis. For example, *NTRK3* was co-regulated by Novel-1187 and Novel-566, as well as another target *PPP1R13L* regulated by Novel-566. These results indicated that these key genes and miRNAs may play an important role in the development of goat hypothalamus and represent candidate targets for further research. This study provides a basis for further explanation of the basic molecular mechanism of hypothalamus, but also provides a new idea for a comprehensive understanding of prolificacy characteristics in Yunshang black goats.

## Introduction

The reproduction is essential to the continuation of every species. The success of reproduction mainly depended on the synthesis and release of hormones. With the release of hormones, a series of reproduction-related events may occur, such as ovulation and fertilization (1). The reproduction of mammals was controlled by the hypothalamic-pituitary-gonadal (HPG) axis regulation network of the reproductive axis. The hypothalamus was not only an important part of the central nervous system, but also the center that regulated animal internal organs and endocrine activities (2). In the reproductive regulatory network, the hypothalamus can receive signals of changes in the internal and external environment, and accordingly regulated the secretion of gonadotropin-releasing hormone (GnRH), thereby regulating the activities of the gonads. The hypothalamus was a key brain region for initiating reproductive activities, which can produce GnRH signals and regulate the secretion of downstream hormones including Follicle-stimulating hormone (FSH) and Luteinizing hormone (LH) (3). Therefore, the hypothalamus played a crucial role in regulating goat reproduction.

With the development of sequencing technology and the application of RNA-seq technology, the miRNA had been discovered as a new type of post-transcriptional regulatory factors, which involved in the biological processes of eukaryotic reproduction and development, as well as cell growth, proliferation, and apoptosis through the post-transcriptional regulation of target genes (4–9). Studies had shown that miRNAs were involved in neurogenesis, neuronal differentiation and migration, and regulated brain function by silencing specific target molecules. For example, Baudry (10) found that miR-16 mediated adult neurogenesis in the hippocampus of rodents. Zhao (11, 12) found that miR-9 and let-7b regulated NSCs proliferation and differentiation by inhibiting the expression of stem cell regulator TLX and its downstream effector molecule, cell cycle regulator *cyclin* D1 in MUS adult NSCs (aNSCs). In addition, miR-19, which played a key role in neuronal migration, had recently been identified. Study (13) had found that miR-19 regulates cell migration by targeting Rapgef2 to regulate the activity of RAP protein, leading to increased migration of newborn neurons in the adult hippocampus. However, the effects of specific miRNAs and their target genes on the development and function of the hypothalamic nucleus and its neuronal lineage remained unclear.

Yunshang black goat was a new breed of meat black goat with excellent traits such as fast growth and high reproduction rate. It was bred after years of cross breeding with Yunling black goat as the female parent and black Nubi goat as the male parent (14).

As the first new breed of black goat for meat in China, Yunshang black goats was of great value to the development of the industry by studying miRNAs and mRNAs related to its lambing number to improve the efficiency of goat reproduction, which has so far not been in-depth about research. Therefore, we chose this species to study the regulatory mechanism of miRNAs and mRNAs in hypothalamic development.

In this research, the hypothalamus of Yunshang black goats in the follicular stage was used as the research object, we analyzed the DEMs and DEGs between FP-HY and FP-LY by RNA-seq, trying to clarify the potential miRNA-mRNA regulatory network affecting fecundity hypothalamic in hypothalamus and identify its key role and the molecular regulation mechanism behind the excellent reproductive performance of Yunshang black goats also as a reference for other mammals.

## Materials and Methods

### Sample Collection and RNA Extraction

It took the ten goats with no significant differences in age, weight, height, body size and body condition were selected as the research object in this study. Additionally, all the goats were bred under the same conditions, with free access to water and feed, in a goat farm of Yunnan province. All selected goats were treated with progesterone vaginal suppository (CIDR) for synchronization of estrus. The vaginal suppository was removed after 16 days of synchronous estrus treatment. The 10 Yunshang black goats, comprising 5 high fertility goats (*n* = 5, individuals with the litter size were more than 2) and 5 low fertility goats (*n* = 5, individuals with the litter size were 1–2) according to their litter size records, were slaughtered within 48 h after CIDR removal in follicular phase. The hypothalamic tissue samples were collected for short-term storage in liquid nitrogen, and then brought back to the laboratory and stored in a refrigerator at−80°C for long-term storage until being used.

Total RNA for RNA-seq was extracted from hypothalamic tissues of 10 Yunshang black goats with TRIzol reagent (Thermo Fisher Scientific, Waltham, MA, United States), and then the Nanodrop 2,000 instrument was used to detect the concentration and purity of the extracted RNA, and the integrity of RNA was detected by agarose gel electrophoresis. The hypothalamic tissue samples of Yunshang black goats in the high fertility group and low fertility group at the follicular stage were marked as FP-HY and FP-LY, respectively.

### Library Construction and Sequencing

A total of 10 cDNA libraries were constructed with the hypothalamic tissues from Yunshang black goats in the follicular phase. The total RNA of 3μg of each sample was used as templates to synthesize complementary DNA (cDNA). Then the obtained double-stranded cDNA was repaired with end, added base A and connected the sequencing adaptors. We used AMPure XP beads to screen cDNA fragments with a length of about 250–300bp, perform PCR amplification, and then used AMPure XP beads to purify the PCR products, and finally obtained the mRNA library. Then, a PE150 (paired-end 150bp, PE150) sequencing approach for mRNAs was performed on the Illumina Novaseq6000 platform.

The Small RNA Sample Pre Kit was used for miRNA library construction for qualified samples. Based on the special structure of 3′ and 5′ ends of small RNA, total RNA was used as the input material. The ends of small RNA were directly spliced, and then reverse transcribed into cDNA. After PCR amplification, the DNA fragment was separated by PAGE gel electrophoresis, and the cDNA library was obtained by gel cutting. Qubit 2.0 and Agilent Bioanalyzer 2100 were used to evaluate the quality and quantity of the cDNA library, and finally the miRNA library was obtained. In addition, a SE50 (single-end 50bp, SE50) sequencing approach for miRNAs was performed on the Illumina Hiseq2500 platform.

### Sequence and Differential Expression Analysis of mRNAs

The raw reads in fastq format were first filtered by the trimming software of SOAPnuke (v2.1.0), using the criteria for removing reads with splice sequences, N bases accounting for more than 1% of reads and low-quality reads. The final reads obtained were clean reads, and then used HISAT2 (v2.1.0) (15) to map the clean reads to the reference genome (GCF_001704415.1). Subsequently, we used String Tie v1.3.5 (16) software to assemble transcripts of mRNA. Used String Tie v1.3.5 to obtain the number of reads compared to each transcript for each sample, and perform FPKM (17) (fragments per kilobase per million bases) conversion to obtain the expression level of the transcript.

After quantification of transcript expression was completed, statistical analysis of its expression data was required and screened transcripts with significantly different expression levels in the sample. We used DESeq2 v3.18.1 (18) to analyze the significance of transcript expression differences. The differentially expressed transcripts were screened from two levels of fold change (FC) and corrected significance level (padj/*q* value). The default setting of the screening threshold was *q* value < 0.05 (If *q* value < 0.05 was used to screen for too few differential genes, then *p-*value < 0.05 was used for differential screening). In addition, |log_2_FC| > 1 and *q* value < 0.05 were considered DEGs in FP-HY vs. FP-LY.

### Sequence and Differential Expression Analysis of miRNAs

Processed the raw sequencing reads, including removing low-quality reads, reads without 3′ adaptor sequences and insert fragment, reads containing continuous A/T/C/G, and final reads with abnormal lengths, and so forth. The following filters were analyzed with clean reads with a length of 18–35nt. Aligned small RNA (sRNA) to the reference genome (GCF_001704415.1) in bowtie, and the comparison of each sample and the distribution of sRNA in the genome were obtained. The Bowtie V1.0.1 (19) was used to align clean reads of each sample with miRNA sequences specified in the mirBase (V22) (20) database to identify the known miRNAs. The sRNAs that were not aligned to the mirBase database were aligned to the Rfam or Repeat Masker database to annotate the ncRNA or repeats sequences of the reference genome. According to the landmark hairpin structure of miRNA precursor, the prediction of the novel miRNAs was carried out based on mirEvo / miRdeep V2.0.0.5 (21). The expression amount of known and novel miRNAs in each sample was counted.

The number of differential miRNAs of the comparison was summarized, and the two levels of fold change (FC) and corrected significance level (padj/*q* value) were evaluated. DESeq2 v3.18.1 was used to identify DEMs in FP-HY vs. FP-LY, and |log_2_FC| > 1, *q* value < 0.05 was considered to indicate differential expression. In addition, the expression amount of the known and novel miRNAs in each sample was counted. Furthermore, MiRanda v3.3a (22) and qTar (https://github.com/zhuqianhua/qTar.git) were used to predict the target genes of miRNAs. In order to ensure the accuracy of the results, the final result was the intersection of the two softwares.

### Gene Ontology and Kyoto Encyclopedia of Genes and Genomes Analyses

The DEGs and the predicted target genes of DEMs were analyzed by Gene Ontology (23, 24) (GO) (referred to as GO, http://www.geneontology.org), which includes biological process, molecular function and cellular component, and Kyoto Encyclopedia of Genes and Genomes (25–27) (KEGG). GO items or KEGG pathways with a hypergeometric *p*-value < 0.05 were those that were significantly enriched.

### Construction of Integral miRNA-mRNA Interaction Networks

The miRNAs had a negative regulation role on the expression of target genes, that was, it could inhibit or degrade the expression of its target genes after transcription. Therefore, the data could be combined and analyzed to perform correlation analysis on DEMs and DEGs, so that to obtain data on the negative regulation of genes by miRNAs in the goat hypothalamus as a whole, and analyze the DEMs and DEGs regulation networks. In order to accurately identify the key association with reproductive DEMs and DEGs, based on miRNA function, the mRNAs that were negatively related to miRNAs were screened out, and the miRNA-mRNA interaction networks were built by using Cytoscape (v3.8.2, http://www.cytoscape.org/).

### Quantitative PCR Validation

In order to validate the accuracy of sequencing data, 6 mRNAs, including *ANAPC15, NAP1L1, FAM91A1, MIER1, SPTAN1* and *AKR1A1*, and 6 miRNAs, including chi-miR-1271-5p, chi-miR-2332, Novel-1461, Novel-1033, chi-miR-125b-3p and chi-miR-34a, were randomly selected for data validation.

For the qPCR analysis of mRNAs, reverse transcription was performed using PrimeScript™ RT reagent kit (TaKaRa, Dalian, China). Furthermore, qPCR with the SYBR Green qPCR Mix (TaKaRa, Dalian, China) was conducted with a RocheLight Cycler®480 II system (Roche Applied Science, Mannheim, Germany) as follows: initial denaturation at 95°C for 5 min, followed by 40 cycles of denaturation at 95°C for 5 s, then annealing at 60°C for 30 s. The sequences of qPCR primers are listed in Table 1.

**Table 1:** The primers of the mRNAs for qPCR.

**Gene name**	**Primer sequence (5^**′**^3^**′**^**	**Product Size (bp)**	**Tm (^**°**^C)**
*AKR1A1*	F: AATGTGGGACCTGGCAAGTT	184	60
	R: GGCTGTCTCCTCGCTCAAAG		
*ANAPC15*	F: CGGCCTCAGAGCACTATGAC	154	60
	R: GGAGGCTCATTGACCTCTCC		
*FAM91A1*	F: TGTATTGCCGATTGGGGTT	139	60
	R: TCCCACGACAGGAGCATCT		
*MIER1*	F: CTCAAGATGCCCAGGAAATAAT	236	60
	R: TAAGTATTCAGGGTCCCACAGG		
*NAP1L1*	F: AGTTCTCAGATGCTGGTCAACC	251	60
	R: AACTGTCCCACGTCCCTTGT		
*SPTAN1*	F: GGAGCTGTACCAGAACCTGAC	255	60
	R: CGCTGGAAAGCAACACTGTC		
*RPL19*	F: ATCGCCAATGCCAACTC	154	60
	R: CCTTTCGCTTACCTATACC		

For the qPCR analysis of miRNAs, reverse transcription was performed using miRcute Plus miRNAs First-Strand cDNA Kit (TIANGEN, Beijing, China). Then, qPCR was conducted miRcute Plus miRNA qPCR Kit (TIANGEN, Beijing, China) using a RocheLight Cycler®480 II system (Roche Applied Science, Mannheim, Germany) in the following procedure: initial denaturation at 95°C for 15 min, followed by 40 cycles of denaturation at 94°C for 20 s, then annealing at 60°C for 34 s. The primers specific to miRNAs were synthesized by Beijing Tianyi Huiyuan Biotechnology Co., Ltd (Beijing, China).

In addition, *RPL19* (for mRNA) and U6 small nuclear RNA (for miRNA) were utilized as reference gene-miRNA to calculate the relative expression level with the method of 2^−Δ*Δct*^. All the qPCR results were presented as the mean ± SD.

## Results

### The cDNA Library Sequencing and Transcriptome Profiles of the Hypothalamus

**Figure 1:**
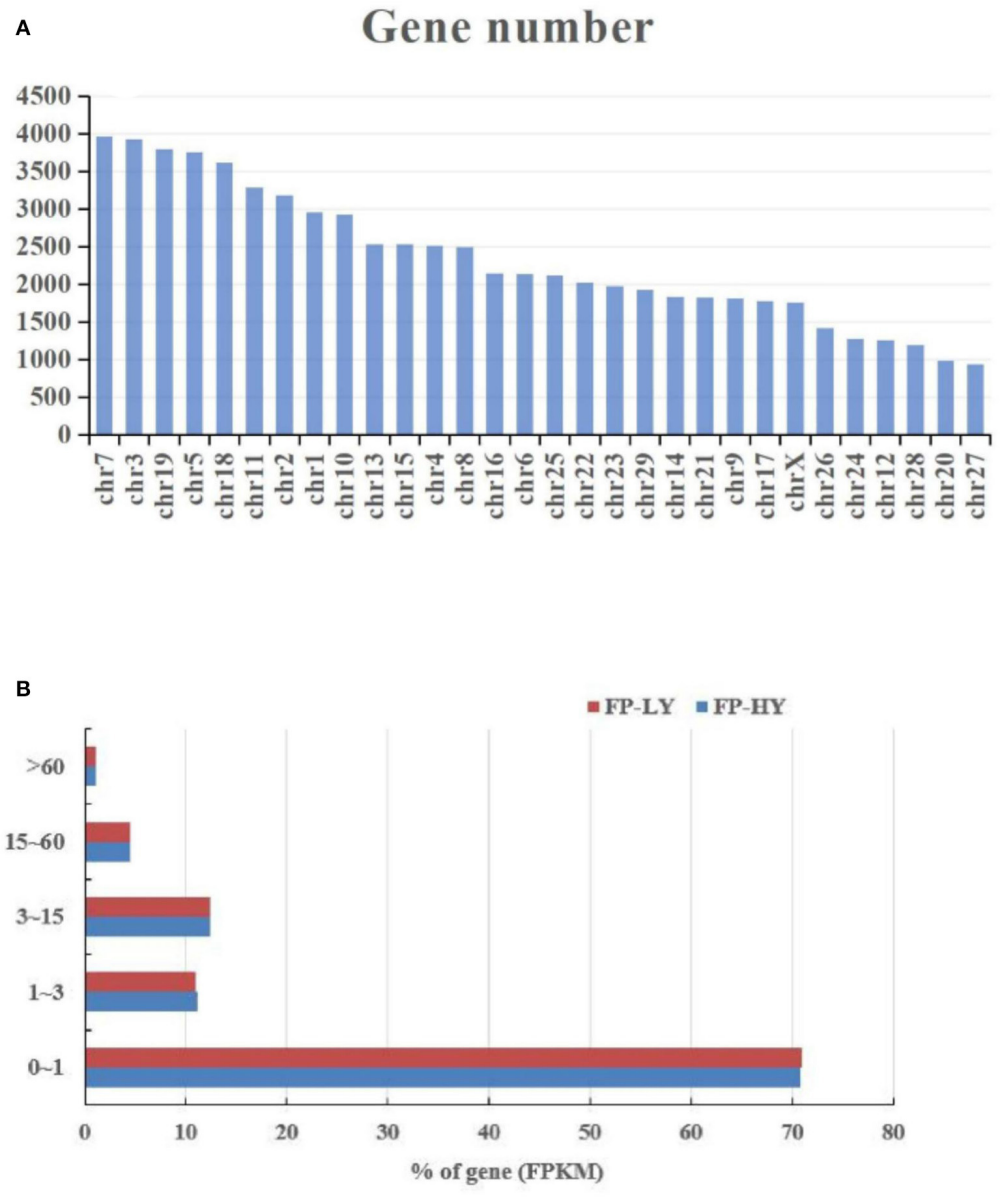
Chromosome **(A)** and FPKM **(B)** distribution of identified genes in FP-HY and FP-LY comparison.

RNA-seq for mRNA generated about 1,119 million clean reads after data filtering, and more than 91.35% of the clean reads were located in the genome. The GC content of clean reads was between 46.1 and 47.9%, and the clean reads quality scores of Q20 and Q30 were above 96.1% and 90.3% respectively, indicating that the reliability and quality of the sequencing data were sufficient for further analysis (Supplementary Table 2). Regarding the expression level of mRNAs, our results showed that RNA-seq highly expressed genes, those with FPKM>60 genes, accounted for about 1.14%, and most genes were distributed between 0 ~ 1 FPKM (Supplementary Table 3, Figure 1A). In addition, the chromosomal distribution of mRNAs showed that chromosome 7 contained 5.67% of hypothalamic genes, followed by chromosome 3 (5.62%) and chromosome 19 (5.43%) (Supplementary Table 3, Figure 1B). Overall, 72,220 mRNAs (Supplementary Table 3) were identified after mapping to the goat genome.

### Small RNA Library Sequencing and miRNA Transcriptome Profiles of the Hypothalamus

**Figure 2:**
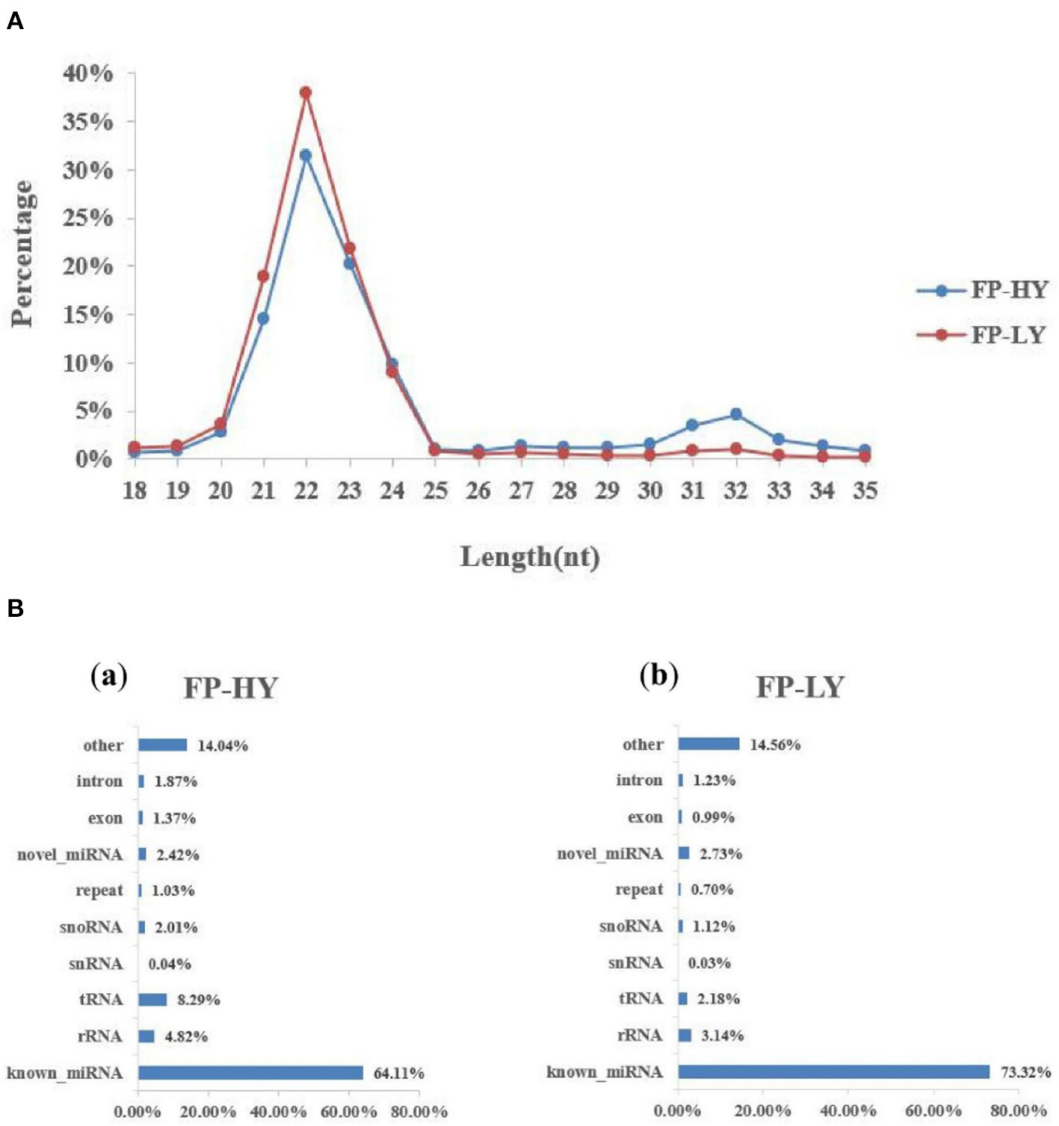
Length distribution of small RNA reads and the percentage of detected miRNAs from NcRNAs. **(A)** Length distribution of clean reads from sRNA fragments. **(B)** Categories of identified non-coding RNAs (NcRNAs) by sequencing in FP-HY (a) and FP-LY (b).

In the sequencing library of hypothalamus samples, a total of 266 million raw reads were obtained. After the raw reads was filtered and processed, about 249 million raw reads reached the quality control standard and were retained as clean reads, with the length ranging from 18 to 35 nt (Supplementary Table 8, Figure 2A). The average Q20 content was 99.16%, and GC content of the clean reads was between 47.75 and 49.23% (Supplementary Table 5). In addition, a variety of non-coding RNAs (ncRNAs) were also identified, including transfer RNAs (tRNAs), snRNAs, miRNAs (Supplementary Table 8, Figure 2B). The known miRNAs accounted for only a small part of all identified ncRNAs. The predicted targeting genes of DEMs were 6,529 (Supplementary Table 9). Finally, a total of 1,837 miRNAs were generated in transcriptome data, including 424 known miRNAs and 1,413 novel miRNAs, which were used in the following analysis (Supplementary Table 6).

### Functional and Pathway Enrichment Analysis of the DEGs Between FP-HY and FP-LY

**Figure 3:**
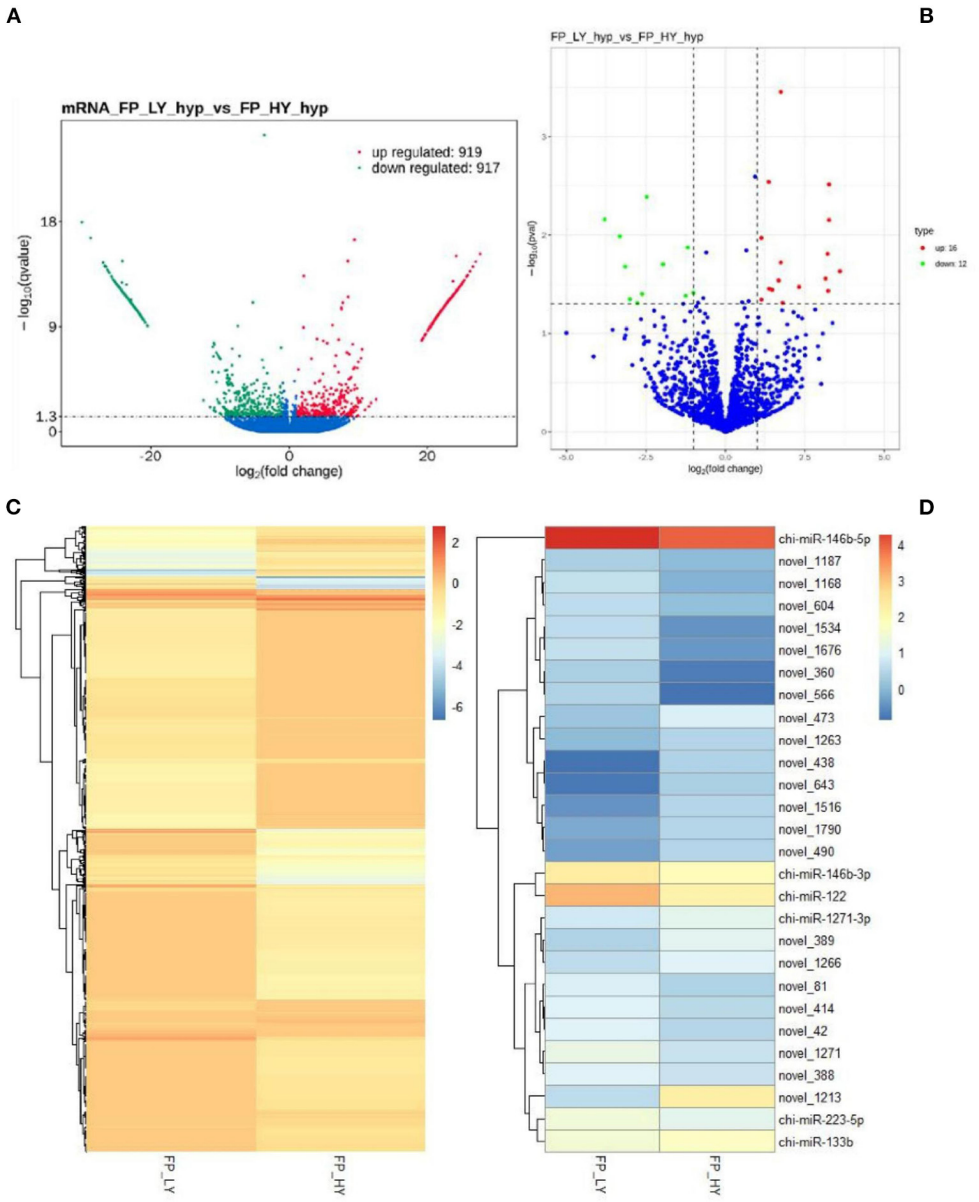
DEGs and DEMs analyses. Volcano plot of identified genes **(A)** and miRNAs **(B)** in FP-HY vs. FP-LY, where red and green represent up- or down-regulation, respectively. Heat maps showing the expression intensity of 1,836 DEGs **(C)** and 28 DEMs **(D)** in FP-HY vs. FP-LY.

**Figure 4:**
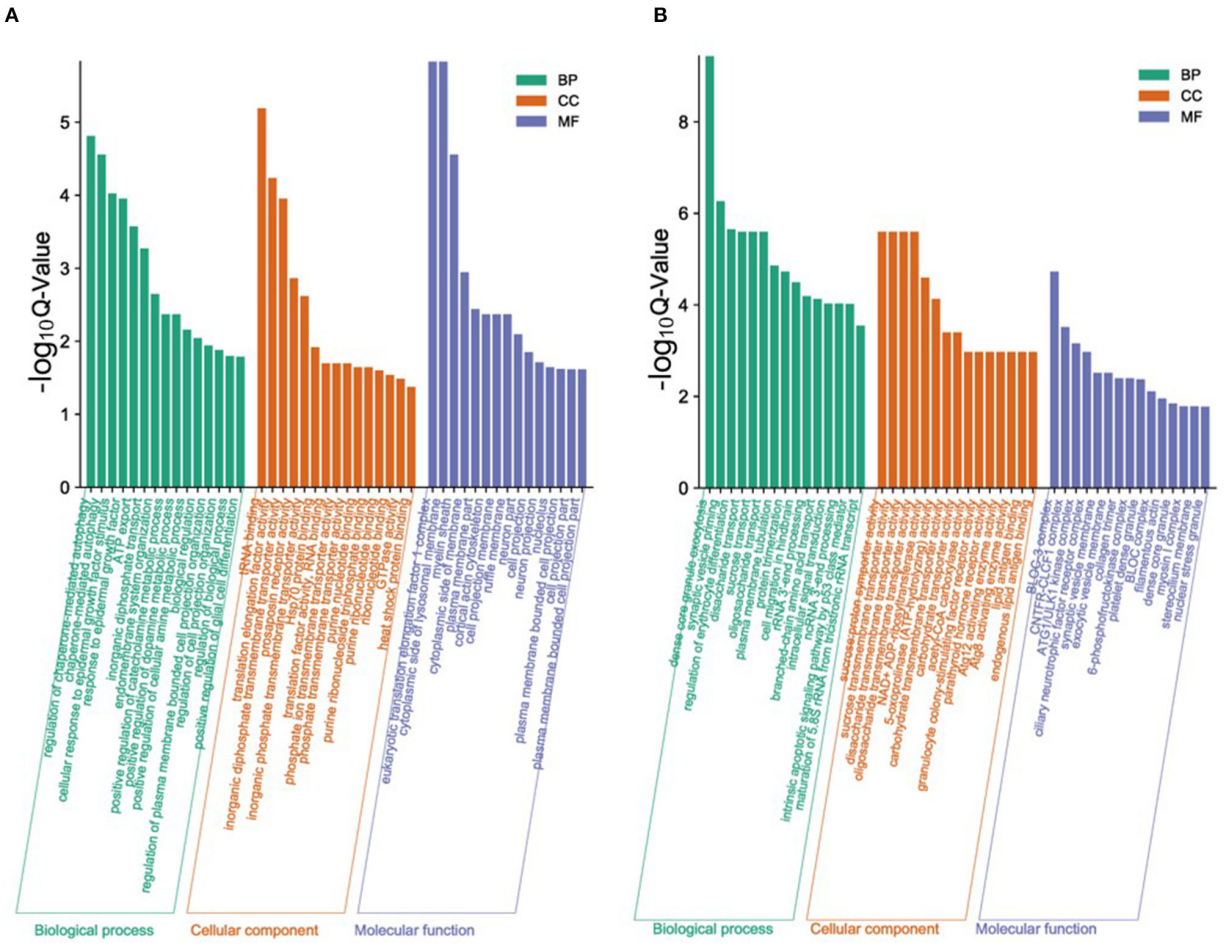
The top 15 enriched GO terms for the identified DEGs and target genes of DEMs in FP-HY vs. FP-LY. Q-Value < 0.05 was used as a threshold to select significant GO terms. The abscissa and ordinate represent the GO terms and the -log_10_Q-Value of enriched genes, respectively. GO, Gene Ontology; BP, biology process. **(A)** GO enrichment terms for DEGs in FP-HY vs. FP-LY. **(B)** GO enrichment terms for target genes of DEMs in FP-HY vs. FP-LY.

**Figure 5:**
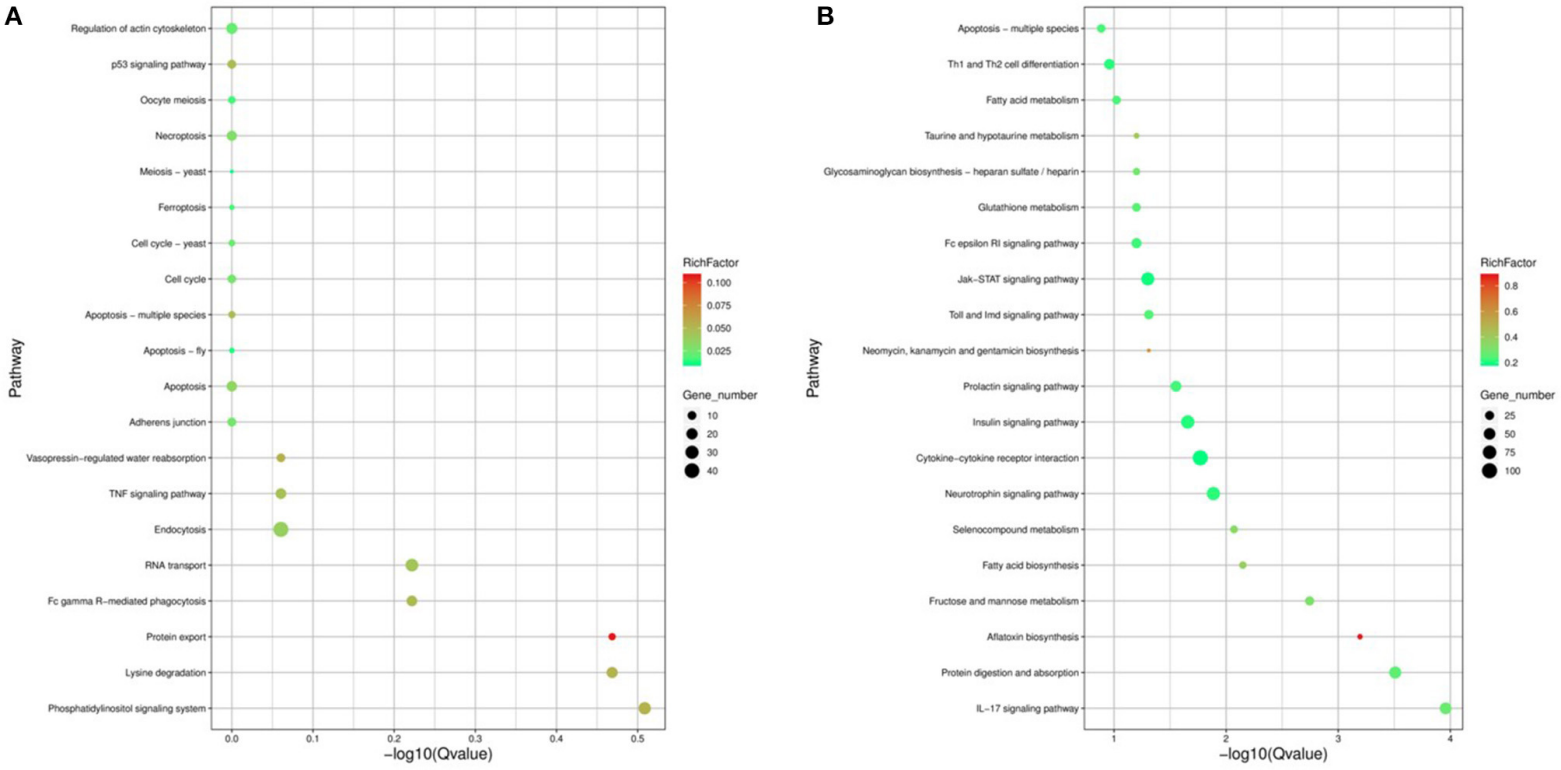
The 20 enriched KEGG for the identified DEGs and target genes of DEMs in FP-HY vs. FP-LY. The abscissa and ordinate represent the -log_10_Qvalue of enriched genes and the KEGG pathways, respectively. Rich factor refers to the ratio of the number of differentially expressed gene and the number of annotation genes enriched in this pathway term. The size of the circle in the figure indicates the number of differential gene enrichment in the pathway. KEGG, Kyoto Encyclopedia of Genes and Genomes. **(A)** KEGG enrichment pathways for DEGs in FP-HY vs. FP-LY. **(B)** KEGG enrichment pathways for target genes of DEMs in FP-HY vs. FP-LY.

In order to study the key genes that affected the number of litters in goat, the DEGs in low- and high-yielding goats were analyzed. A total of 1,836 DEGs were identified from FP-HY vs. FP-LY comparison. Among these DEGs, 919 genes were up-regulated and 917 genes were down-regulated (Supplementary Table 4, Figure 3A). The DEGs showed different expression patterns between FP-HY and FP-LY (Figure 3C). To further understand the biological functions of DEGs, we performed GO and KEGG enrichment analysis on 1,836 differentially expressed mRNAs in the follicular phase of the hypothalamus. In GO terms, the most enriched terms in FP-HY and FP-LY are Eukaryotic Translation Elongation Factor 1 Complex (GO: 0005853) and Cytoplasmic Side of Lysosomal Membrane (GO: 0098574) (Supplementary Table 10, Figure 4A). KEGG enrichment analysis revealed that DEGs was significantly enriched in 8 KEGG pathways (Supplementary Table 11, Figure 5A), of which the most enrichment pathway was Phosphatidylinositol signaling system (ko04070), the signal transduction system may play a role in the reproductive, which need to be further studied.

### Functional and Pathway Enrichment Analysis of the DEMs Between FP-HY and FP-LY

A total of 28 DEMs were detected in 1,837 identified miRNAs (Supplementary Table 7). Among them, 16 miRNAs were up-regulated and 12 mRNAs were down-regulated (Table 2, Figure 3B). In addition, DEMs showed a significantly different expression pattern between FP-HY and FP-LY (Figure 3D). In order to better understand the biological functions of the 28 identified DEMs, we predicted the potential target genes of these miRNAs. There were 1,280 and 5,903 target genes predicted in 6 known and 22 novel DEMs from FP-HY and FP-LY comparison, respectively. Some putative target genes were repeated.

**Table 2:** 28 DEMs in the hypothalamus of Yunshang black goats between FP-HY and FP-LY.

**sRNA**	**FPKM (FP-LY)**	**FPKM(FP-HY)**	**Log_**2**_(FC)**	**q-value**	**Down/Up**
novel-1213	179.71	3.38	−5.74	0.0002	Down
chi-miR-146b-5p	9162.23	30768.96	1.75	0.0003	Up
chi-miR-146b-3p	78.43	202.22	1.36	0.0029	Up
chi-miR-122	144.62	1386.59	3.26	0.0031	Up
novel-473	7.01	1.48	−2.48	0.0041	Down
novel-1263	2.65	0.00	−3.80	0.0069	Down
novel-1534	0.33	3.74	3.26	0.0071	Up
novel-1516	2.81	0.32	−3.32	0.0103	Down
chi-miR-223-5p	11.49	25.45	1.13	0.0107	Up
chi-miR-1271-3p	12.92	5.47	−1.19	0.0134	Down
novel-1676	0.36	3.78	3.22	0.0155	Up
novel-1271	4.72	15.94	1.75	0.0189	Up
novel-389	10.19	2.54	−1.97	0.0198	Down
novel-438	2.60	0.13	−3.15	0.0209	Down
novel-1187	0.00	2.09	3.61	0.0233	Up
novel-360	0.18	2.19	3.15	0.0276	Up
novel-81	2.30	7.60	1.68	0.0290	Up
novel-1168	0.80	3.79	2.32	0.0338	Up
novel-414	3.15	7.99	1.38	0.0352	Up
novel-42	2.82	8.18	1.47	0.0361	Up
novel-566	0.15	2.43	3.23	0.0370	Up
chi-miR-133b	55.11	27.83	−1.01	0.0386	Down
novel-1790	2.63	0.64	−2.62	0.0398	Down
novel-1266	8.35	3.57	−1.25	0.0415	Down
novel-643	2.25	0.17	−3.00	0.0449	Down
novel-388	4.43	9.98	1.13	0.0453	Up
novel-604	1.14	3.73	1.80	0.0489	Up
novel-490	2.90	0.47	−2.77	0.0492	Down

In GO analysis, the top 2 enriched terms were dense core granule exocytosis (GO:1990504) and synaptic vesicle priming (GO:0016082) (Figure 4B, Supplementary Table 12). KEGG analysis (Supplementary Table 13, Figure 5B) showed that the most abundant pathway is IL-17 signaling pathway (ko04657). In addition, the Jak-STAT signaling pathway (ko04630) and cytokine-cytokine receptor interactions (ko04060) had also been enriched. Through GO and KEGG pathway analysis, many pathways and GO terms were associated with reproduction. Among them, 34 KEGG pathways were identified as significantly related to the DEMs targeting genes. All of these pathways are related to the basic activities of life, such as the prolactin signaling pathway, the Jak-STAT signaling pathway, and the GnRH signaling pathway.

### MiRNA-mRNA Interaction Network

**Figure 6:**
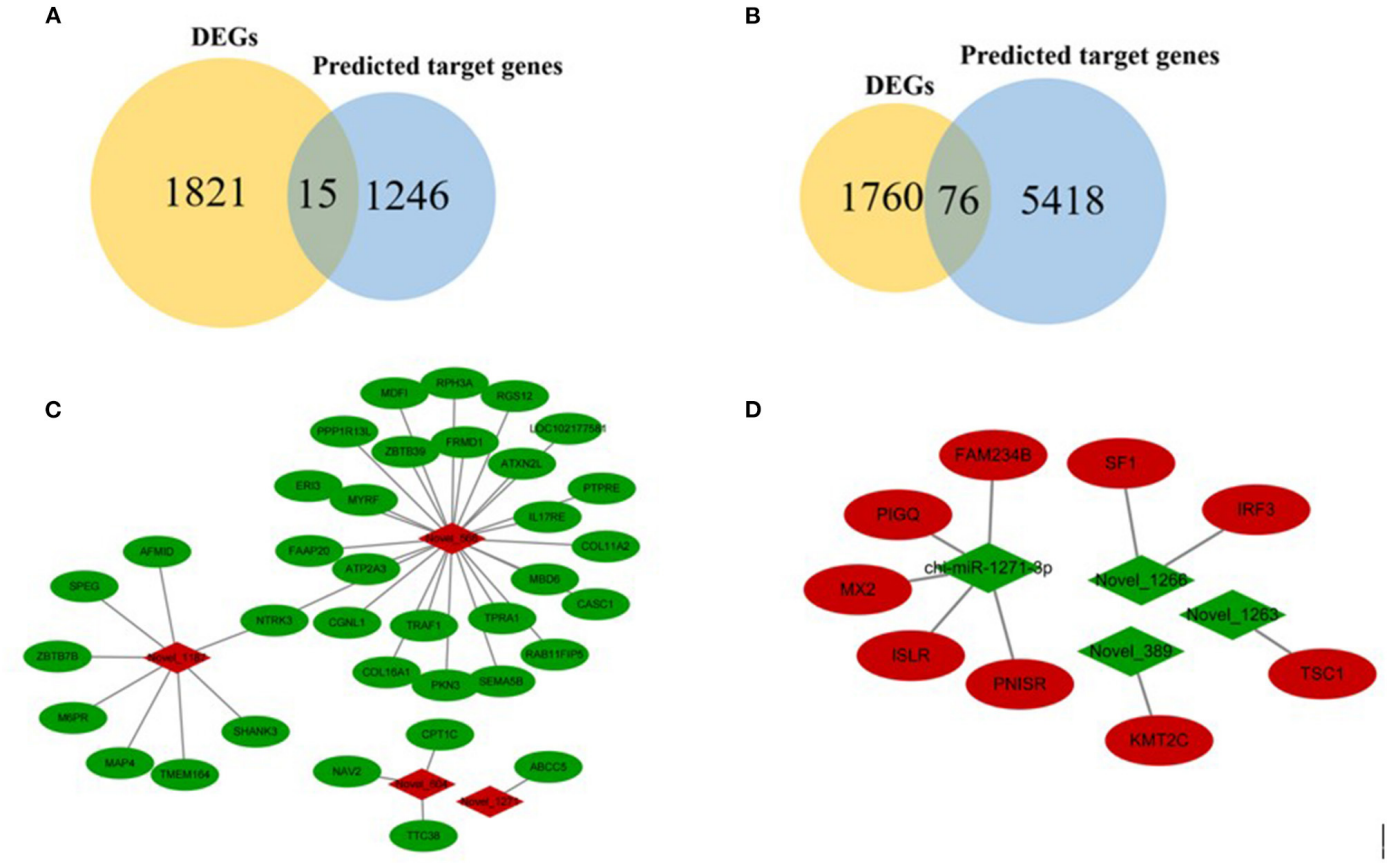
Overview of mRNA-miRNA networks. **(A)** Intersected genes in FP-HY vs. FP-LY between DEGs and predicted target genes by known miRNAs. **(B)** Intersected genes in FP-HY vs. FP-LY between DEGs and novel miRNA-targeted genes. **(C)** Main hypothalamic upregulated network in FP-HY vs. FP-LY containing the 4 upregulated miRNAs and 36 target genes. **(D)** Main hypothalamic downregulated network in FP-HY vs. FP-LY containing the 4 downregulated miRNAs 4 and 9 target genes. Red and green indicate up- or down-regulation, respectively.

In order to further analyze the relationship between miRNAs and mRNAs, we used DEMs and their target genes (DEGs) to construct an interactive network. The potential targeting genes of the 28 identified DEMs were intersected with 1,836 DEGs obtained from RNA-seq, and 87 intersected genes (IGs) were obtained (Supplementary Table 14, Figures 6A,B). Then Cytoscape (28) was used to construct an interactive network of miRNA-mRNA pairs (Supplementary Table 15). The main upregulated miRNA-mRNA interactive network indicated that 36 DEGs were targeted by 4 DEMs, of which 4 novel miRNAs (Figure 6C). The main downregulated miRNA-mRNA interactive network showed that 9 DEGs were targeted by 4 DEMs, including 1 known miRNA and 3 novel miRNAs (Figure 6D).

### Data Validation

**Figure 7:**
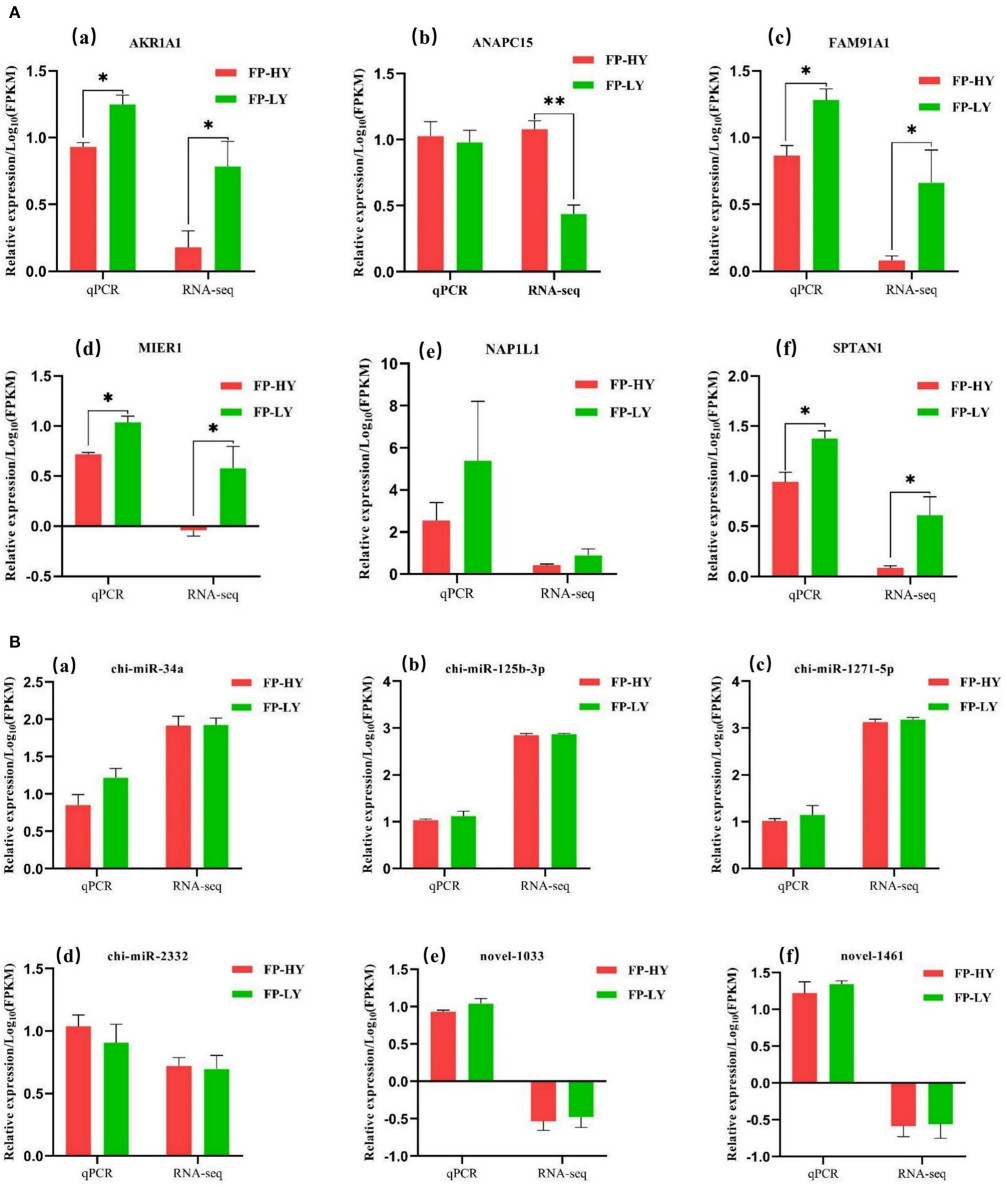
qPCR validation of mRNAs **(A)** and miRNAs **(B)** identified by RNA-seq in FP-HY and FP-LY. **p <0.01, *p < 0.05.

In order to assess the accuracy of sequencing, mRNAs and miRNAs were selected randomly for qPCR validation. We measured the gene expression level and compared it with the RNA-seq data. The results demonstrated that RNA-seq data and qPCR data showed similar patterns (Figure 7), which indicating the reliability of the data generated from RNA-seq.

## Discussion

The number of goatling was a very important reproductive trait in goats. It was a trait controlled by minor effects and polygenes. It was not only affected by nutritional level and environment, but also by heredity (29). At present, many signal pathways had been unearthed through molecular biological technology research and were involved in the occurrence of goat reproduction (30), but its genetic and physiological mechanisms need to be further explored. The hypothalamus was an important nerve center that regulates the body and endocrine activities, as well as an important center of neuroendocrine. It can regulate the secretion of hormones in the pituitary gland and the activities of its target glands, including reproduction and growth (31). Here, we initially identified 1,836 DEGs and 28 DEMs from FP-HY vs. FP-LY comparison in the hypothalamus by RNA-seq.

In this study, we found that miR-9 and miR-7 were the most highly expression both in the FP-HY and FP-LY groups of hypothalamus, which was accordance with the results of studies in humans (32) and goats (33). In addition, we also identified the miRNA family members including chi-let-7 and chi-miR-200 in FP-HY vs. FP-LY. And study had found that the DEMs identified in seasonal and non-seasonal sheep ovaries contain these two family members (34), but the miR-200 family members were in mice (35), rats (36) and goats (in this study) had no differential expression. This research proved that a variety of miRNAs were expressed in the hypothalamus of goats, and had different expression levels in goats with different fertility. However, some miRNAs showed species specificity, which may cause differences in reproductive phenotypes between different animals. Hypothalamic miRNAs were mainly involved in reproductive development processes by regulating GnRH neurons. However, it may be that the research was difficult and the types of miRNAs involved were few, so related studies had focused on their targets.

## Functional Analysis of DEGS in FP-HY vs. FP-LY

In the functional enrichment analysis of DEGs, we found several key genes including Bone Morphogenetic Protein Receptor 1B (*BMPR1B*), Fibroblast Growth Factor Receptors 1 (*FGFR1*), Insulin-like Growth Factor 1 (*IGF1*) and cAMP Response Element Binding Protein 1 (*CREB1*) were involved in the reproductive process. *BMPR1B* was one of the candidate genes for high fecundity that had been paid more attention nowadays. Study had shown that BMPR1B was a key molecule in the BMP signaling pathway, which regulated and affected the number of ovulation and littering of sheep (37). Growth Differentiate Factor 5 (*GDF5*) and Bone Morphogenetic Protein 4 (*BMP4*) were the natural ligands of *BMPR1B*. The expression of *BMPR1B* was regulated by these two proteins, which pathway had an impact on sheep reproduction (38).

*FGFR1* was a tyrosine kinase receptor, which can activate a series of downstream pathways by binding to fibroblast growth factor to regulate embryonic development, wound healing (39). The *FGFR1* gene also regulated the migration of GnRH nerves, which was essential for neuron development and embryonic reproductive system development (40, 41). Our results showed that the expression of *FGFR1* in FP-HY was significantly higher than that in FP-LY, and the expression of *GnRH1* was significantly higher in FP-LY than in FP-HY. Therefore, we speculated that there may be a negative regulatory relationship between *FGFR1* and *GnRH1* in the goat hypothalamus.

*IGF1*, as a hormone regulating factor, can directly act on GnRH neurons, and that cooperated with GnRH to promote follicular growth and steroid hormone synthesis to regulate reproductive activities (42, 43). In terms of growth regulation, it mainly promoted cell metabolism together with GH (growth hormone), and promoted growth and regulated reproduction (44). *CREB* was the common nuclear target of extracellular signal-regulated kinase (ERK) signaling pathway and PI3K signaling pathway (45). Study had shown that the activation of PKA/CREB was the key to regulating GnRH synthesis in GnRH neurons (46), and *CREB1* was involved in the regulation the expression of *FSH*β gene (47). Activated *CREB1* activated target gene transcription, thereby regulating the differentiation of germ cells (48). In addition, *CREB1* also controlled the response of nerve cells to extracellular stimuli, such as regulating *IGF-1* at the transcriptional level (49). Our results showed that the expression of *IGF1* in FP-HY was significantly lower than that in FP-LY, while *CREB1* showed the opposite expression pattern between FP-HY and FP-LY. Therefore, we can speculate that there may be a negative regulatory relationship between *IGF1* and *CREB1* in goat hypothalamus.

In general, these 4 genes played an important role in the regulation of animal reproduction. The biological function of DEGs was a candidate function to explain the differences in reproduction among the analyzed animals. In addition, these DEGs were mainly concentrated in several KEGG pathways, including TNF signaling pathways, Jak-STAT signaling pathway, metabolic pathways (50), neural signal transduction pathways, and pathways related to biological systems and cellular processes. These pathways played a vital role in the biosynthesis of reproductive hormones. And study had shown that the Jak-STAT signaling pathway had important functions in GnRH neurons, thereby participating Reproductive process (51). We hypothesized that these pathways may regulate the transcriptional expression of genes related to reproduction by participating in the communication between neurons.

## Analysis of miRNA-mRNA Interaction Network

### Analysis of miRNA-mRNA Interaction Network in FP-HY vs. FP-LY

In order to better understand the functions of miRNAs, we constructed a miRNA-mRNA interaction network in FP-HY vs. FP-LY. The up-regulated network contains 36 mRNAs and 4 miRNAs negative interactors, and the down-regulated network contains 9 mRNAs and 4 miRNAs.

In the up-regulated network, Neurotrophic tyrosine receptor kinase 3 (*NTRK3*) was the common targeting gene of Novel-1187 and Novel-566. This gene was a member of the NTRK family, and NT-3 was its ligand. Its main function was to regulate cell proliferation, differentiation, and apoptosis (52–54). The function of NTRK3 is ligand-dependent, which promoting cell growth and differentiation in the presence of ligands and inducing apoptosis in the absence of ligands (55). At the same time, another targeting gene of Novel-566, *PPP1R13L*, was the only anti-apoptotic member in the P53 apoptosis stimulating protein of p53 family (ASPP). When *PPP1R13L* was highly expressed, which can inhibit the function of *P53* gene to induce apoptosis (56). In addition, Mannose 6-phosphate receptor (M6PR), another target gene of novel-1187, was a multifunctional protein which can interact with a variety of ligands and participated in the regulation of cell growth and apoptosis (57). Some of these ligands were important growth regulators, such as Insulin-like growth factor-II (IGF-II) (58). M6PR affects the internalization and lysosomal degradation of IGF-II, which usually stimulates cell proliferation through IGF-I receptor (59). In conclusion, we infered that DEMs may further affect the hormonal activity related to reproduction by affecting the apoptosis of nerve cells and ultimately lead to differences in goatling number.

In the down-regulation network, tuberous sclerosis complex (*TSC1*) was the targeting gene of Novel-1263 and was found to participate in the PI3K/Akt/TSC1-TSC2/mTOR signaling pathway to regulate cell transcription and translation, autophagy, and apoptosis (60). Steroidogenic Factor 1 (*SF1*) was reversely regulated by Novel-1266, which belonged to the nuclear receptor superfamily, and was a key regulator of steroid production and reproductive function in mammals (61, 62). Besides, the immunoglobulin superfamily containing leucine rich repeat (ISLR) gene, which was a target gene of chi-miR-1271-3p, had been identified as a novel marker for mesenchymal stem cells and expressed in stem cells,. Study had found that ISLR can affect the activation of the canonical Wnt signaling pathway by targeting Dishevelled-2 (Dvl2) (63). The Wnt signaling pathway was involved in the formation of the brain (64). Therefore, the research on ISLR was very necessary and important. We speculated that DEMs affect signal pathways related to reproduction by regulating target genes, which may further affect litter size.

The results of this study showed that several key DEGs and DEMs in the hypothalamus were directly or indirectly involved in hormonal activities related to reproduction. Further research on mRNA/miRNA knockdown or overexpression will help us understand their real function in goat reproductive traits.

## Conclusion

This research provides the first complete mRNA-miRNA network in Yunshang black goats from the perspective of the hypothalamus in the follicular stage. We identified several DEGs (*BMPR1B, FGFR1, IGF1*, and *CREB1*) and miRNA-mRNA pairs (*NTRK3* is a targeting gene of Novel-1187 and Novel-566) from the RNA-seq data obtained from goat hypothalamus. These miRNAs and genes may play an important role in the regulation of the hypothalamus in the follicular phase, which was worthy of further study on the reproductive regulation of goat prolificacy mechanism in the future study.

## Data Availability Statement

The datasets presented in this study can be found in online repositories. The names of the repository/repositories and accession number(s) can be found below: https://www.ncbi.nlm.nih.gov/sra/?term=PRJNA755189.

## Ethics Statement

The animal study was reviewed and approved by Institute of Animal Sciences and Chinese Academy of Agricultural Sciences (IAS-CAAS) (Beijing, China) for all the experimental procedures mentioned. Ethical approval was provided by the Animal Ethics Committee of IASCAAS (No. IAS2019-63). Written informed consent was obtained from the owners for the participation of their animals in this study.

## Author Contributions

MH and MC: conceptualization. MH: data curation, formal analysis, and writing—original draft. MH, CL, ZZ, YL, XH, YJ, YO, QH, and MC: investigation. MH, ZZ, YL, and MC: methodology. QH, YJ, YO, and MC: resources. MH and YL: software and supervision. MH and ZZ: validation. CL and MC: writing—review and editing. CL, QH, and MC: project administration and funding acquisition. All authors have read and agreed to the published version of the manuscript.

## Funding

This work was financially supported by China Agriculture Research System of MOF and MARA (CARS-38), the Science and Technology Innovation Talents Program of Yunnan Province (2018HC017), the Basic Research Foundation Key Project of Yunnan Province (202001AS070002), Agricultural Science and Technology Innovation Program of China (CAAS-ZDRW202106 and ASTIP-IAS13), Key Research and Development Project of Shanxi Province (201903D211008), Shanxi Province 1331 Project Key Disciplines of Animal Sciences (J201911301), and Shanxi Agricultural University Doctoral Research Initiation Project (2021BQ04).

## Conflict of Interest

The authors declare that the research was conducted in the absence of any commercial or financial relationships that could be construed as a potential conflict of interest.

## Publisher's Note

All claims expressed in this article are solely those of the authors and do not necessarily represent those of their affiliated organizations, or those of the publisher, the editors and the reviewers. Any product that may be evaluated in this article, or claim that may be made by its manufacturer, is not guaranteed or endorsed by the publisher.
