# Supplementary material for: Hypothalamic Transcriptome Analysis Reveals the Crucial MicroRNAs and mRNAs Affecting Litter Size in Goats
Source: Front Vet Sci. 2021 Nov 1;8:747100. doi: 10.3389/fvets.2021.747100 (PMC8591166; doi:10.3389/fvets.2021.747100)
Supplement: Supplementary file 1 [file Table_1.DOC]

**Table S2. Statistics for the cDNA library sequences of Yunshang black goats hypothalamic tissue.**

| Items | Clean Reads | Mapped Reads | Mapping Ratio | Q20 | Q30 | GC_content |
| --- | --- | --- | --- | --- | --- | --- |
| FP-HY1 | 98,851,856 | 90,305,919 | 91.35% | 96.1 | 90.3 | 47.0 |
| FP-HY2 | 109,799,302 | 105,607,482 | 96.18% | 98.3 | 95.0 | 47.1 |
| FP-HY3 | 104,936,226 | 101,362,792 | 96.59% | 98.4 | 95.1 | 46.1 |
| FP-HY4 | 115,269,140 | 111,876,562 | 97.06% | 98.6 | 95.6 | 46.7 |
| FP-HY5 | 123,907,622 | 120,217,348 | 97.02% | 98.6 | 95.0 | 47.0 |
| FP-LY1 | 108,005,280 | 104,787,818 | 97.02% | 98.6 | 95.1 | 47.9 |
| FP-LY2 | 122,059,690 | 118,228,643 | 96.86% | 98.6 | 95.6 | 46.7 |
| FP-LY3 | 110,033,906 | 106,673,054 | 96.95% | 98.5 | 95.5 | 47.8 |
| FP-LY4 | 114,911,814 | 111,131,014 | 96.71% | 98.6 | 95.7 | 46.4 |
| FP-LY5 | 110,828,462 | 106,724,217 | 96.30% | 98.4 | 95.1 | 46.6 |
