# Supplementary material for: Hypothalamic Transcriptome Analysis Reveals the Crucial MicroRNAs and mRNAs Affecting Litter Size in Goats
Source: Front Vet Sci. 2021 Nov 1;8:747100. doi: 10.3389/fvets.2021.747100 (PMC8591166; doi:10.3389/fvets.2021.747100)
Supplement: Supplementary file 4 [file Table_4.DOC]

**Table S5. Statistics for the small RNA library sequences of Yunshang black goats hypothalamic tissue.**

| Items | Raw  Reads | Clean  Reads | Clean  Ratio | Mapped  Reads | Mapping  Ratio | Q20 | GC_content |
| --- | --- | --- | --- | --- | --- | --- | --- |
| FP-HY1 | 27,261,095 | 26,313,834 | 96.53% | 25,564,252 | 97.15% | 99.31% | 48.37% |
| FP-HY2 | 30,426,805 | 28,225,848 | 92.77% | 27,111,468 | 96.05% | 99.07% | 48.64% |
| FP-HY3 | 26,747,383 | 25,570,084 | 95.60% | 24,796,398 | 96.97% | 99.09% | 48.76% |
| FP-HY4 | 24,148,217 | 21,826,316 | 90.38% | 20,945,221 | 95.96% | 99.22% | 49.14% |
| FP-HY5 | 28,227,650 | 26,437,202 | 93.66% | 25,889,565 | 97.93% | 99.31% | 48.43% |
| FP-LY1 | 28,054,967 | 25,694,789 | 91.59% | 25,114,918 | 97.74% | 99.26% | 49.07% |
| FP-LY2 | 22,916,650 | 22,315,579 | 97.38% | 21,669,360 | 97.10% | 98.74% | 47.88% |
| FP-LY3 | 28,290,090 | 27,295,346 | 96.48% | 26,707,369 | 97.85% | 99.32% | 47.75% |
| FP-LY4 | 21,823,555 | 19,327,787 | 88.56% | 18,856,728 | 97.56% | 99.21% | 49.23% |
| FP-LY5 | 27,820,830 | 26,040,540 | 93.60% | 25,312,099 | 97.20% | 99.11% | 48.70% |
