# Supplementary material for: Hypothalamic Transcriptome Analysis Reveals the Crucial MicroRNAs and mRNAs Affecting Litter Size in Goats
Source: Front Vet Sci. 2021 Nov 1;8:747100. doi: 10.3389/fvets.2021.747100 (PMC8591166; doi:10.3389/fvets.2021.747100)
Supplement: Supplementary file 15 [file Table_15.DOC]

Supplementary Material

**Supplementary Table S2 |** Overview of the quality control of mRNA reads generated from hypothalamic tissues

**Supplementary Table S3 |** The overall genes expressed and the fragments per kilobase per million mapped fragments (FPKM) values and chromosomes distribution of identified mRNAs identified in high fecundity goats in the follicular phase (FP-HY) versus low fecundity goats in the follicular phase (FP-LY).

**Supplementary Table S4 |** Differentially expressed genes identified in high fecundity goats in the follicular phase (FP-HY) versus low fecundity goats in the follicular phase (FP-LY).

**Supplementary Table S5 |** Overview of the quality control of miRNAs reads generated from the hypothalamic tissues.

**Supplementary Table S6 |** The overall miRNAs expressed identified in high fecundity goats in the follicular phase (FP-HY) versus low fecundity goats in the follicular phase (FP-LY).

**Supplementary Table S7 |** Differentially expressed miRNAs identified in high fecundity goats in the follicular phase (FP-HY) versus low fecundity goats in the follicular phase (FP-LY).

**Supplementary Table S8 |** The identification involving diverse RNAs and length distribution of small RNA in high fecundity goats in the follicular phase (FP-HY) versus low fecundity goats in the follicular phase (FP-LY).

**Supplementary Table S9 |** The list of potential target genes of differentially expressed miRNAs in high fecundity goats in the follicular phase (FP-HY) versus low fecundity goats in the follicular phase (FP-LY).

**Supplementary Table S10 |** GO enrichment annotation for mRNAs in terms of their molecular function (MF), biological process (BP), and cellular component (CC) level in high fecundity goats in the follicular phase (FP-HY) versus low fecundity goats in the follicular phase (FP-LY).

**Supplementary Table S11 |** KEGG enrichment annotation for mRNAs in high fecundity goats in the follicular phase (FP-HY) versus low fecundity goats in the follicular phase (FP-LY).

**Supplementary Table S12 |** GO enrichment annotation for potential target genes of differentially expressed miRNAs in terms of their molecular function (MF), biological process (BP), and cellular component (CC) level in high fecundity goats in the follicular phase (FP-HY) versus low fecundity goats in the follicular phase (FP-LY).

**Supplementary Table S13 |** KEGG enrichment annotation for potential target genes of differentially expressed miRNAs in high fecundity goats in the follicular phase (FP-HY) versus low fecundity goats in the follicular phase (FP-LY).

**Supplementary Table S14 |** The intersected gene list between DEGs and predicted target genes of DEMs in high fecundity goats in the follicular phase (FP-HY) versus low fecundity goats in the follicular phase (FP-LY).

**Supplementary Table S15 |** Construction of miRNA-mRNA Interaction Network in high fecundity goats in the follicular phase (FP-HY) versus low fecundity goats in the follicular phase (FP-LY).
